# Incorporating neurological and behavioral mechanisms of sociality into predator-prey models

**DOI:** 10.3389/fnbeh.2023.1122458

**Published:** 2023-04-17

**Authors:** James L. L. Lichtenstein, Oswald J. Schmitz

**Affiliations:** ^1^Department of Biology, Kenyon College, Gambier, OH, United States; ^2^Yale School of the Environment, Yale University, New Haven, CT, United States

**Keywords:** functional responses, trait plasticity, social behavior, social dominance, intraspecific competition, mechanistic predation models

## Abstract

Consumer-resource population models drive progress in predicting and understanding predation. However, they are often built by averaging the foraging outcomes of individuals to estimate per capita functional responses (functions that describe predation rate). Reliance on per-capita functional responses rests on the assumption that that individuals forage independently without affecting each other. Undermining this assumption, extensive behavioral neuroscience research has made clear that facilitative and antagonistic interactions among conspecifics frequently alter foraging through interference competition and persistent neurophysiological changes. For example, repeated social defeats dysregulates rodent hypothalamic signaling, modulating appetite. In behavioral ecology, similar mechanisms are studied under the concept of dominance hierarchies. Neurological and behavioral changes in response to conspecifics undoubtedly play some sort of role in the foraging of populations, but modern predator-prey theory does not explicitly include them. Here we describe how some modern approaches to population modeling might account for this. Further, we propose that spatial predator-prey models can be modified to describe plastic changes in foraging behavior driven by intraspecific interaction, namely individuals switching between patches or plastic strategies to avoid competition. Extensive neurological and behavioral ecology research suggests that interactions among conspecifics help shape populations’ functional responses. Modeling interdependent functional responses woven together by behavioral and neurological mechanisms may thus be indispensable in predicting the outcome of consumer–resource interactions across systems.

## Introduction

A core goal of community ecology is to build mechanistic and predictive understanding of consumer-resource dynamics (Sutherland et al., 2013). Managers might use this understanding, for example, with managed fish populations and the plankton they eat, to predict the rise and fall of the fish populations (Hunsicker et al., 2011; Hacini et al., 2021). Predictive consumer-resource models would then inform the choice of management strategies that sustain harvestable fish population size or promote its stability. A key element of modeling approaches that aim to predict consumer-resource population dynamics is the functional response. The functional response relates per capita consumer foraging rates to resource abundance (Holling, 1959; DeLong, 2021; Krebs, 2022). Functional responses come in several functional forms from being a constant by which we multiply the population densities of predator and prey to get a predation rate, but they are often more elaborate functions (DeLong, 2021; Abrams, 2022). These more elaborate versions of functional responses have been a primary means by which different aspects of predator prey biology are incorporated into theoretical ecology (Real, 1977; Ajraldi et al., 2011; Dawes and Souza, 2013; Abrams, 2022). For instance, Holling’s disk equation (often referred to as the Type ii functional response) began efforts to incorporate biological mechanisms, in this case predators taking refractory time from hunting to consume their prey (Holling, 1959, 1966). A rich literature has followed to account for other mechanisms in functional response models (Abrams, 2022; Krebs, 2022), which we will continue to do here. Specifically, we will consider evidence from the behavioral and neurosciences suggesting that intraspecific interactions among consumers can cause individuals functional responses to be interdependent (functions whose shape depend on each other).

Our examination here addresses the core assumption that a per capita function can reliably relate consumer and resource density to the rate of resource consumption. We examine a way to overcome complications that arise from the implicit schism between two distinct methods for constructing functional responses: measuring the feeding behavior of individuals or of populations (Duijns et al., 2015; Griffen, 2021). These methods ostensibly measure the same process, but the individual-level approach models functional responses as a characteristic of individuals—which modelers multiply by the number or biomass of individuals, and the population-level approach which treats functional responses as a characteristic of populations. The way variation among individuals is treated in the different methods stems directly from differences in how they are calculated. This generates profound differences in how functional responses are conceptualized and the kinds of predictions that arise for consumer-resource populations.

At the population level, functional responses are measured by observing how consumer and resource population sizes change over time and space, and using these data to build models of the relationship between predator and prey density (Gill et al., 2001; Goss-Custard et al., 2006; Gillings et al., 2007; Smart et al., 2008). This approach relies on then extrapolating the functional parameters measured for a population across time and contexts. The population-level functional response perspective ultimately ends up being an exercise in curve fitting natural dynamics and comparing which models fit the data best (Krebs, 2022). Insight about biological mechanisms is resolved through the observation of natural feeding behavior. For example, by incorporating observations of red knots (*Calidris canutus*) switching among foraging patches and incorporating site switching mechanisms into their models, van Gils et al. (2015) were able to construct functional response models that better predicted prey depletion. By directly observing population-level processes, this approach can realistically estimate functional response parameters, to the extent that these parameters are constant across time and space. However, these population-level responses still describe a function that simply scales the typical individual to population-level processes.

At the individual level, functional responses are measured by observing how readily multiple individual predators consume prey at different prey densities, often in laboratory settings (Holling, 1966; Griffen, 2021). Toscano and Griffen (2014) built functional responses to account for variation in the rate individual crabs consume mussel prey at different densities. This approach relies on estimating the average response across multiple, individual predators to build models of the relationship between prey and predator densities. This views functional responses as something that can characterize an individual consumer’s response, which is somehow related to its foraging traits (DeLong, 2021). Indeed, traits related to consumer feeding biology determine the shape of functional responses (Schröder et al., 2016; DeLong et al., 2021). Hence, more explicit linkages between individual foraging traits and response are called for to resolve individual-level mechanisms (Lima and Dill, 1990; Kalinoski and DeLong, 2016). For example, individual crabs with a propensity to be more active eat more mussels at high mussel densities than less active individuals (Toscano and Griffen, 2014). Thus it might be better to think of functional responses as phenomena that emerges from the traits of consumer and resource species and thereby subject to evolution, something akin to the “soft traits” described by Hodgson et al. (1999).

**FIGURE 1:**
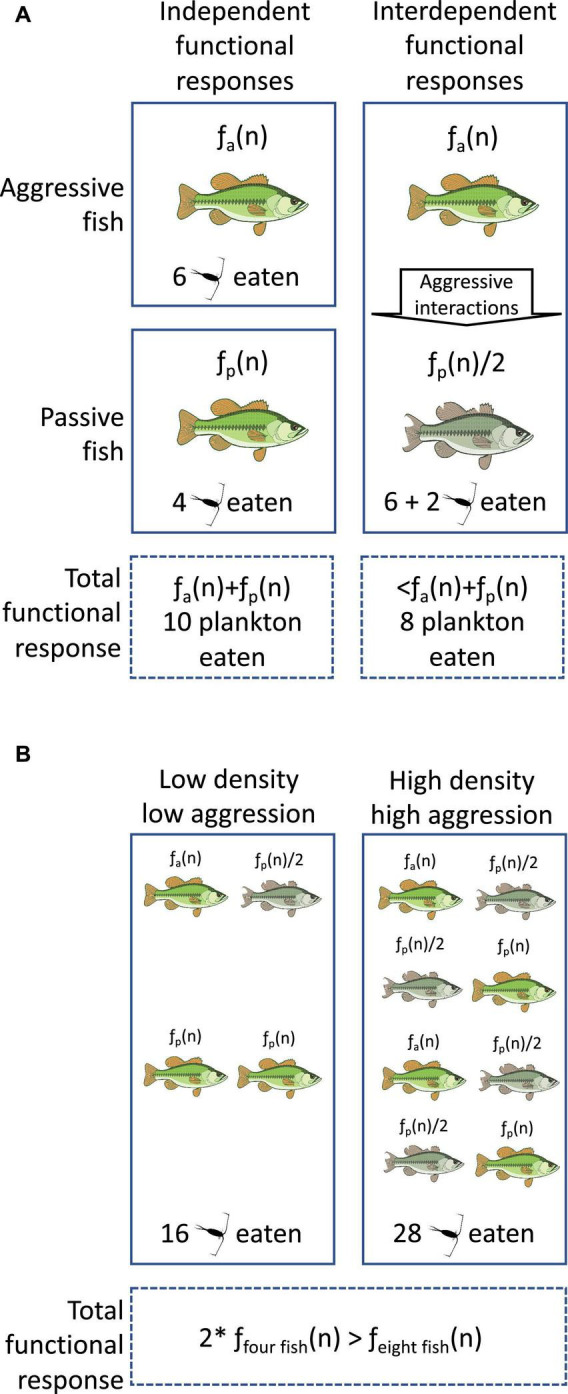
The individual- and population-level consequences of interdependent functional responses. Fish eat plankton with the *per capita* functional response, f(n). Aggressive and passive fish have different functional responses before any aggression. **(A)** In the same pond as aggressive fish, the stress of aggression halves the functional responses of passive fish, decreasing how many plankton are eaten compared to the two fish separately. **(B)** At the population level, increased density increases the rate at which aggressive fish reduce the functional responses of passive fish. Thus, doubling the density of fish and holding constant the ratio of aggressive to passive fish does not double the number of plankton eaten. *Means multiplied by.

How then do these individual- and population-centered methods of measuring functional responses compare? When measured in tandem, individual-derived and population-derived functional responses do not always produce the same estimates (Duijns et al., 2015). This difference might be reconciled by considering how functional responses vary plastically across different environments (DeLong et al., 2014, 2023; Wang et al., 2022). For consumers, that environment is often other members of their own species (Hurd and Eisenberg, 1984; Relyea, 2002). Feeding behavior, and thereby functional responses, can be very sensitive to interactions with conspecifics (Toyoda, 2017; Natterson-Horowitz and Cho, 2021). Hence, when measuring functional response by isolating consumers experimentally, such as in aquaria *sensu* Toscano and Griffen (2014), either assumes individual consumers act in isolation of other individuals in a population. If they do not act in isolation, then the practice of modeling predation as proportional to predator density will inaccurately predict predator population dynamics by ignoring important among-individual interactions (Figure 1). We highlight here that individuals can affect the functional responses of each other by: (1) denying through aggressive behavior access to food and (2) using this aggressive behavior to trigger plastic changes in feeding behavior in the targets of this aggression. Much current predator prey-theory does not account for interactions among consumer individuals and so assumes consumer individuals operate independently (as opposed to theory incorporating interactions among prey; Ajraldi et al., 2011; Djilali, 2019; Ghanbari and Djilali, 2020). Extensive work in behavioral ecology and neuroscience challenges this theoretic assumption. Taking behavioral mechanisms, like the following, into account will increase the accuracy of predator-prey models (Lima, 2002; Hacini et al., 2021; Abrams, 2022).

## Individuals’ functional responses can constrain or expand conspecific functional responses

Populations of any species often include individuals more disposed to antagonistic vs. neutral or cooperative interactions (Sih and Watters, 2005; Eldakar et al., 2009). This can manifest as conspecific aggression and cannibalism (Sih et al., 1998). Some fishing spiders (*Dolomedes triton*) are more prone to cannibalism than others for example (Johnson and Sih, 2005). Further, there are salmonid fish individuals that are more prone to aggressive interactions their entire lives (Nicieza and Metcalfe, 1999). As well, some evidence suggests that aggression and sociality or cooperation might also form a plastic continuum through underlying neurogenetic mechanisms, suggesting that some individuals more than others readily switch between being aggressive/social and not (Kelly and Vitousek, 2017). Yet the tendency toward among-individual aggression and cannibalism often depends on resource availability (Fox, 1975; Hurd and Eisenberg, 1984). Hence members of any population may at different times or in different locations plastically switch between aggressive vs. social tendencies. We develop the thesis here that these tendencies mechanistically link how individuals capture their own prey to how they influence other population members’ capture of their prey.

Much work on animal aggressiveness has featured consistent differences in individuals’ tendencies to attack conspecifics (Drent et al., 1996; Bell et al., 2009). Some of this work has tested whether tendencies to attack conspecifics correlate with tendencies to attack prey (David et al., 2011; Chang et al., 2017). For example, fishing spiders that more readily attack prey their whole lives were more likely to attack potential mates as adults (Johnson and Sih, 2005). More aggressive salmon ate more and grew faster than passive salmon, but aggressive salmon tended to attack other aggressive individuals more so than their passive counterparts (Nicieza and Metcalfe, 1999). This evidence suggests that individuals with stronger functional responses (those that eat more prey in general) lean toward the aggressive end of the distribution of conspecific interaction tendencies.

How then does this aggression by individuals with strong foraging responses affect conspecific functional responses (when the targets of aggression are not eaten)? The long-term consequences of patterns of aggression may become most manifest in cases with dominant and subordinate status differences. Individuals who are habitually aggressive toward conspecifics over resources tend to be referred to as socially dominant, and the targets of that aggression are referred to as socially subordinate (Drews, 1993). Dominant individuals often have more access to food, and restrict subordinate individuals’ access to food through aggressive behavior (Natterson-Horowitz and Cho, 2021). In several fish species, whenever dominant individuals are kept together with subordinate individuals, the dominants tend to gain weight more quickly than submissive (Jobling and Wandsvik, 1983; Koebele, 1985; Maclean and Metcalfe, 2001). Further, the dominant individuals can decrease subordinate feeding by instigating subordinate avoidance behavior, a case of cryptic interference competition (Gyimesi et al., 2010; Ferry et al., 2016). Among foraging red knots, dominant individuals spent more time foraging than subordinate individuals even though very few actually aggressive interactions occurred (Bijleveld et al., 2012). Status hierarchies can be easily observed in some species, but are often plastic and poorly defined in others (Milewski et al., 2022). Status nonetheless represents a mechanism by which the functional responses of individuals can be interdependent. How the functional responses of subordinate individuals are constrained could be conceptualized similarly to how competition constrains fundamental niches into realized niches.

These interactions cost one individual, but they benefit the other. Similar outcomes can emerge without aggression in the case of producer scrounger-dynamics, wherein some individuals put effort into finding food, whereas others follow in their wake looking to capitalize on their success. Wild baboons were more likely to capitalize on the foraging opportunities of less dominant females in patches with high food availability (King et al., 2009). This can be observed even in facultatively social species (Vickery et al., 1991; Evans et al., 2021). Again, animals can often (but not always, see Groothuis and Carere, 2005; Kurvers et al., 2010) switch strategies, and they have been found to converge on frequency-based optima for given environmental contexts (Giraldeau et al., 1994; Mottley and Giraldeau, 2000). How would the frequency of producers or scroungers in a population affect the shape of individual and population level functional responses, especially when their success is frequency dependent? This is an older, well-studied, foraging mechanism which ecological theory on consumer-resource interactions has yet to explicitly consider.

Sociality creates alternative outcomes. Interactions among social consumers can allow individuals to expand each other’s feeding ability (Sih et al., 1998; Schmitz, 2007), potentially mutually strengthening individuals’ functional responses (Nilsson et al., 2007; Přibylová and Peniašková, 2017; Sen et al., 2019). Social foraging is the prime example of this phenomenon and it has been studied exhaustively, occurring in a wide array of taxa from social arthropods (Hölldobler and Wilson, 1990; Avilés, 1997) to many vertebrate groups (Wilson, 2000). Social animal functional responses are often measured at a population level (Theberge, 1990; Hayes and Harestad, 2000), or individual level responses are measured for social individuals in isolation (Fritz et al., 2001; Latifian et al., 2018). What does the functional response of an individual ant or wolf in isolation even mean? Overall, despite overwhelming interest in the ecological impacts of obligately socially foraging animals (Wilson, 1990; Mech and Boitani, 2007; Boyer et al., 2010), social foraging has scarcely been applied to functional response models (Nilsson et al., 2007; Djilali and Ghanbari, 2021). Social foraging entails a spectrum of complexity and integration, ranging from egrets facultatively corralling fish (Wiggins, 1991), to the extensive obligate agricultural behavior of some ant species (Mueller et al., 1998; Schultz and Brady, 2008). In contrast to this paucity of theory, a fair amount of work has gone into how the social behavior of prey might increase their survival (Ajraldi et al., 2011; Djilali, 2019; Ghanbari and Djilali, 2020; Hacini et al., 2021; Brahim et al., 2022). Predator-prey ecology has only begun to work on small parts of this vast spectrum, and complex interactions can emerge even in less social species.

This literature offers promising support for earlier theoretical ideas that the functional responses of any given species might be inclined to change the shape of other individuals’ functional responses (Anders, 2001; Nilsson et al., 2007). The scope of available evidence, however, is insufficient to make general claims that individual functional responses are likely always interdependent in populations. In the wake of mechanisms such as cryptic interference competition (Gyimesi et al., 2010) that have not been widely studied, it may also not be safe to assume the functional responses of any consumer species are independent. Such interactions—whether antagonistic or social—could generate a wide range of cryptic effects by generating plastic neurological responses in feeding behavior. These could have legacy effects that persist in the absence of conspecifics (Toyoda, 2017; Natterson-Horowitz and Cho, 2021). Hence, it is time to begin exploring the behavioral and neurological mechanisms that drive these individual tendencies, how they influence individual’s capture of their own prey and how they influence other population members’ capture of their prey.

## Social interactions can cause long-term neuroplastic changes in feeding behavior

We have outlined above how aggression and sociality alter functional responses by constraining or expanding what individuals can do on short time scales. However, there is also evidence aggressive and social interactions can trigger longer-term plastic changes in subordinate individuals’ behavior (Natterson-Horowitz and Cho, 2021). Some of the most mechanistically detailed evidence comes from research on social defeat, an animal model of depression (Toyoda, 2017). Social defeats in the form of staged contest between two animals of the same sex, can decrease or increase the appetite and weight of mice (Krishnan et al., 2007; Goto et al., 2014), rats (de Jong et al., 2005), and hamsters (Foster et al., 2006). This suggests that negative social interactions can change feeding behavior and thus functional responses, while also providing mechanistic insight into how this might work.

In rats, social defeat and the resulting decreased feeding behavior was associated with increased levels of hypothalamic Malonyl CoA (Iio et al., 2012, 2014). Malonyl CoA is a key coenzyme and substrate of fatty acid metabolism, and in mammalian hypothalami it also may play a role in appetite suppression (Wolfgang and Lane, 2008). Perturbations of this mechanism can result in appetite suppression that can last as long as 10 days but often subsides by 30 days (de Jong et al., 2005; Krishnan et al., 2007). Hence, appetite responses have a comparable time scale of effect to the social stress itself (very often 10 days, Toyoda, 2017). Further, positive interactions between familiar rats decreases this recovery time (de Jong et al., 2005). This aligns with research that social subornation and dominance can be plastic (Milewski et al., 2022).

However, additional evidence suggests there is a heritable component to this plastic mechanism. Some inbred mouse strains seem to vary considerably in their susceptibility to social defeat stress responses (Toyoda, 2017). One particularly anxious strain decreased its weight gain in response to chronic social stress, whereas a less anxious strain gained weight in response to social defeat (Savignac et al., 2011). Social defeat studies suggest that being attacked shifts rodents into states of altered appetite that positive social interactions can reverse via hypothalamic Malonyl CoA metabolism. Further, this suggests that their populations might contain considerable variability in not only the sensitivity of the neurological response, but also the direction of response. These mechanisms could serve as a jumping off point for studying the evolution of functional response interdependence in mammals.

These illustrative case studies speak to the nature but not the ubiquity of these plastic changes. Again, drawing from literature on social dominance across vertebrate taxa reveals that socially dominant individuals not only limit the ability of subordinate individuals to acquire resources, but they seem to cause changes similar to those seen in studies of social defeat (Natterson-Horowitz and Cho, 2021). For instance, social stress has been found to trigger dramatic weight loss in multiple livestock species (Treasure and Owen, 1997). Fish decrease their growth rate while at lower social ranks, but increase their growth rate once they increase in social rank, in several species (Buston, 2003; Wong et al., 2008). These responses could be yet even more widespread, but much work does not distinguish whether weight loss in subordinate individuals stems from food deprivation directly or plastic responses (Natterson-Horowitz and Cho, 2021).

Collectively, these studies suggest that positive and negative social interactions could have complex effects on the feeding behavior, and thereby on the functional responses of many animals. For instance, having a high density of rats could increase the occurrence of social defeats, but increased access to friendly conspecifics might ameliorate this, depending on shifting contexts. However, there are some important caveats here. First many of these studies are performed in simplified captive environments. Captive environments lacking in sensory and social stimulation can manifest depression-like symptoms in captive environments (Alexander et al., 1978; Simpson and Kelly, 2011). Second, rats and mice are especially social animals (Lacey et al., 2007), so social defeat might have different evolutionary significance than it would for an animal that does not rely on close relations with group members for survival. Hence it provides another mechanism for how interdependent functional responses might emerge. Exploring the mechanism of switching between diet states might be particularly useful in demystifying what these results mean for functional responses.

## Consequences of interdependent functional responses and potential solutions

The next challenge is using understanding of non-independence of functional responses to inform new conceptualizations of how these facets should be included. We first note that in some species of consumers, individuals’ functional responses may not noticeably affect each other. Predator species that migrate far and live at very low densities, such as large pelagic sharks or medusazo, might be good examples. However, their social interactions may just be poorly studied (Findlay et al., 2016). But this underscores that species vary widely in how conspecifics communicate and interact with each other (Wilson, 2000; Searcy and Nowicki, 2010), leading to considerable variation in how interdependent their functional responses are.

### Individual-level consequences

The interdependence of functional responses compromises our ability to justify simply measuring the functional responses of predators in isolation of other predators and make inferences about average effects in populations. For example, we could measure the average individual-level functional responses of fishery raised juvenile salmon on wild macro-invertebrates. However, because more socially dominant salmon tend to suppress the appetite and eating behavior of more subordinate salmon (Nicieza and Metcalfe, 1999), the captive estimates will not equal the fish’s wild functional response. Hence simply averaging across all individuals will give an inaccurate representation of the average population level functional response (Figure 1). Interdependence might help explain why individual- and population-level functional responses do not match (Duijns et al., 2015). Trusting functional responses measured on individuals might be fraught without information on the intraspecific interactions any species performs (Arditi and Ginzburg, 2012; Griffen, 2021).

Increasing work has examined functional responses in terms of individual trait variation (Fox and Murdoch, 1978; Schröder et al., 2016; Toscano et al., 2020). These efforts have been hamstrung by the immense sample sizes demanded by testing how individuals traits affect the classic method of fitting individual level responses based on the feeding rates of many individuals (Holling, 1959; Coblentz and DeLong, 2021). Recent advances, however, allow for functional responses to be measured for single individuals. By observing how quickly a single individual can “eat down” a large number of prey, their attack rate at difference prey densities can be observed (Coblentz and DeLong, 2021). This greatly reduces the labor required to measure the functional responses of individuals, paving the way to testing how all these individual functional responses fit together.

One promising approach is using non-linear averaging of individual functional responses. This method accounts for how the modality in the distribution of individual responses can bias population level responses using Jensen’s inequality (Coblentz et al., 2021). Otherwise, population estimates made with conventional averaging will overestimate how much predators will eat (Ruel and Ayres, 1999; Bolnick et al., 2011). Functional response interdependence could be characterized as non-linearity in the relationship between predator density and how much their population eats (Nilsson et al., 2007). Increasing the density of predators increases how many predators are eating prey, but it also increases interactions among conspecifics and thus non-independence of functional responses (Fox, 1975; Hurd and Eisenberg, 1984). This makes the effect of predator density on prey consumption non-linear (Figure 1B). Non-linear estimation could be used to incorporate non-linearity that arises from functional response interdependence into population models. These lines of research can incorporate the interdependence of functional responses into parameter estimation, and provide a path forward for individual-level functional responses.

### Population-level consequences

Functional response interdependence has even more subtle consequences for population-level measurements of functional responses. The validity and predictive ability of population level functional responses depend on how stable parameter estimates are across time and space (Krebs, 2022). Parameters measured by observing population dynamics of many individuals inherently may include how individual-level functional responses combine to form a population level response (Duijns et al., 2015). These mechanisms are implicitly built into these estimates when they drive real predator–prey interactions, but they are not explicitly detailed by models.

The problem here is that all of the mechanisms of functional response interdependence are plastic and variable across time. The proportions of individuals with aggressive vs. social tendencies in a population will fluctuate across time due to plasticity or selection (Dingemanse et al., 2004; Duckworth et al., 2015). Further, dominance hierarchies and other forms of social interactions themselves often change across different environmental conditions (Herbers and Banschbach, 1999; Block and Stoks, 2004; Milewski et al., 2022). Population-level functional responses rely on their parameters being consistent across time. The interdependence of functional responses, especially when it involves long term plastic changes, could undermine the stability and reliability of these parameters. If a functional response is measured during high resource availability and those resources grow scarce, individuals might increasingly antagonize each other and change each others’ functional responses. This would shift the population functional response away from the high resource estimate. Without modeling that change, the population functional response estimates could rapidly expire.

For this reason, many modeling approaches have already tried to characterize interference among conspecific consumers (Skalski and Gilliam, 2001). Interference is implied and characterized by scaling the functional response to a per population size effect, which then causes resource consumption to change non-linearly with consumer density. The most prominent models among them are ratio-dependent and Beddington-DeAngelis response models (Arditi and Ginzburg, 1989; Skalski and Gilliam, 2001). These models are subtly different and have been debated fiercely (Abrams and Ginzburg, 2000). Both can be derived from mechanisms other than predator interference. The Beddington-DiAngelis model can be derived from predator-prey interactions as opposed to predator-predator interactions (Huisman and De Boer, 1997; Geritz and Gyllenberg, 2012). Ratio-dependent models can be reproduced using first principles by modeling predator–prey interactions in terms of limitation by energy rather than time (Schoener, 1973). Although they are not necessarily based in predator interference, they can approximate these mechanisms because they predict that predator density would increase or decrease the rate at which individual predators kill.

This is a subtle distinction, but it is crucial. When these models are fit to real data, predator-dependent models can fit consumer-resource population dynamics better than models that do not account for predator density (Skalski and Gilliam, 2001; Novak et al., 2017). However, different models perform better or worse for different populations, but the reason is not clear (Skalski and Gilliam, 2001; Novak et al., 2017). Without a modeling approach that more explicitly accounts for interference mechanisms, we will be left to merely to compare the fit of different approximation curves and try to parse meaning from the exercise. Further, given the plasticity of these mechanisms, the best model is given to change across time. For predictive predator prey ecology to move forward, mechanisms of functional response interdependence must be incorporated not just into measurements of functional responses, but also their models.

### How to model functional response interdependence

The interdependence of functional responses can cause the functional responses of individuals to be plastic across environmental gradients of interaction intensity with members of their own species (Fox and Murdoch, 1978; Thompson, 1978; Wang et al., 2022; DeLong et al., 2023). Yet, few modeling approaches incorporate parameter plasticity explicitly (DeLong et al., 2014).

A modeling approach that incorporates non-linear dynamics should thus include how these parameters can be plastic across some range of environmental conditions.

We suggest doing this by coopting another form of consumer-resource model: a two-site spatially implicit predator-prey model. These models account for how the ability of predator and/or prey to move across different foraging patches affects predator- prey interactions (Dos Santos and Costa, 2010; Kang et al., 2017). They assign different values for predator-prey model parameters for different patches, such that the population sizes of predators/prey at different patches depend on the movement of predators, prey or both among sites and the predator-prey interactions at each site (Huang and Diekmann, 2001). We propose using “patches” and the functions that describe movement among them to model changes among consumer plastic “states” as described by work on social defeat and social dominance (Toyoda, 2017; Natterson-Horowitz and Cho, 2021).

This is a functional response approach based on the neurological mechanism of appetite dysregulation by malonyl-CoA (Iio et al., 2012, 2014). We adapt the models proposed by Kang et al. (2017) where terms for migration are added to Rosenweig-McArthur models. In our model predators use a shared pool of resources, but alternate across different states via a function driven by interactions among consumers:


$$\frac{d\,V}{d\,T}=r\,V\,\left(1-\frac{V}{k}\right)-\frac{a_{i}\,P_{i}\,V}{1+h_{i}\,V}$$

$$\frac{d\,P}{d\,T}=\frac{c\,a_{i}\,P_{i}\,V}{1+h_{i}\,V}-\delta \,P_{i}+P_{j}\,a_{j}\,m_{j\,i}-P_{i}\,r_{i}$$

$$a_{i}<a_{j}$$

Here, *V* and is the density of a resource species, *r* is their intrinsic growth, *k* is their carrying capacity, then *P* is the density of a consumer species, a is their attack rate, *h* is their handling time across the stressed state *i* and the unstressed state *j*, *c* is their assimilation efficiency, *δ* is their mortality rate, *m*_*ji*_ is the movement constant from state *j* to state *i*, *r*_*i*_ is the rate at which individuals recover on their own. Research on plastic responses to intraspecific aggression suggests that consumers should also leave distressed states at a certain rate without re-exposure (de Jong et al., 2005; Krishnan et al., 2007). Assimilation efficiency and mortality rate could very well vary across states, but for the sake of simplicity, we only have consumer density and functional responses vary across states. Functional response interdependence is incorporated by having the attack rate of unstressed state *j* drive movement into stressed state *i* at rate *m*_*ji*_, then move back at rate *r*_*i*_. This is the most basic possible function for movement among states driven by functional responses, and more elaborate functions that include more mechanisms for movement among states could certainly be developed. For example, how social interactions increase the rate at which individuals leave stressed state *i* could be added.

This approach is based on just one mechanism of functional response interdependence. There are undoubtedly more generalizable ways to model functional response interdependence. Approaches that model plasticity based on a continuum rather than discrete states would be especially interesting. Any method will likely benefit from non-linear averaging (Coblentz et al., 2021). This approach would be well suited to modeling how consumers are sampled from discrete states or a distribution of plastic responses as they encounter each other and resource species. However, the use of any interdependent functional response approach depends on detailed knowledge of how much exactly the assumption of functional response independence holds up in any consumer population.

## Conclusion

We have offered a substantial body of evidence suggesting that the functional responses of any individual consumer might affect those of other members of their species. The evidence is not widespread enough that we can assume functional responses are always interdependent, but it is abundant enough that we cannot assume that functional responses are independent. Moreover, direct empirical work on functional response interdependence is needed here. When the functional response of individuals are interdependent, it undermines the assumption that consumer populations have a per capita functional response that we can multiply by the density of consumers to get an estimate of how much they eat. We do not mean to imply that previous studies on functional responses are thus invalid. Instead, we argue that functional response approaches need to evolve to incorporate this prevalent facet of animal biology. This could be expanded further to look at social interactions among prey species (Djilali, 2019; Ghanbari and Djilali, 2020; Hacini et al., 2021). If we aim to use ecological modeling to predict consumer-resource interactions, we must continue the push toward including mechanistic facets of their biology. Increasing the range of mechanisms ecological modeling can use will only strengthen the practice (Abrams, 2022).

## Author contributions

Both authors jointly developed the ideas included in this review, contributed to the writing process, and contributed to the article and approved the submitted version.
